# Oleanolic Acid Exerts a Neuroprotective Effect Against Microglial Cell Activation by Modulating Cytokine Release and Antioxidant Defense Systems

**DOI:** 10.3390/biom9110683

**Published:** 2019-11-01

**Authors:** José M. Castellano, Silvia Garcia-Rodriguez, Juan M. Espinosa, María C. Millan-Linares, Mirela Rada, Javier S. Perona

**Affiliations:** Department of Food and Health, Instituto de la Grasa-CSIC, Campus of the University Pablo de Olavide, Building 46, 41013 Seville, Spain; jmcas@ig.csic.es (J.M.C.); silvia11_12@hotmail.com (S.G.-R.); spinnosa@gmail.com (J.M.E.); mcmillan@ig.csic.es (M.C.M.-L.); mrada@ig.csic.es (M.R.)

**Keywords:** oleanolic acid, microglia, neuroprotection, oxidative stress, cytokines, inflammation

## Abstract

Microglia respond to adverse stimuli in order to restore brain homeostasis and, upon activation, they release a number of inflammatory mediators. Chronic microglial overactivation is related to neuroinflammation in Alzheimer’s disease. In this work, we show that oleanolic acid (OA), a natural triterpene present in food and medicinal plants, attenuates the activation of BV2 microglial cells induced by lipopolysaccharide (LPS). Cell pretreatment with OA inhibited the release of IL-1β, IL-6, TNF-α, and NO, which was associated with the downregulation of the expression of genes encoding for these cytokines and inducible nitric oxide synthase (iNOS), and the reinforcement of the endogenous antioxidant cell defense. These findings advocate considering OA as a novel neuroprotective agent to inhibit oxidative stress and inflammatory response in activated microglia associated with Alzheimer’s disease.

## 1. Introduction

In 2017, between 45 and 50 million individuals suffered from Alzheimer’s disease (AD), and it is expected that the prevalence of the disease will quadruple by 2050. AD, the most common form of dementia, is characterized pathologically by a substantial neuronal and synaptic loss, chronic inflammation, and a decrease in cognitive abilities [1]. The causes of AD are not fully understood, but it is well known that abnormal deposits of amyloid-β (Aβ) and neurofibrillary aggregates of hyperphosphorylated tau protein occur in the cerebral cortex [2]. These events worsen with age, and also include inflammation, oxidative stress, and mitochondrial dysfunction [3,4]. The presence of plasma Aβ in the brain is consistent with a weakening of the blood-brain barrier (BBB) integrity. Thus, extravasation of circulating neuroinflammatory molecules may increase the onset risk and worsen the development of the disease [5].

Emerging evidence indicates that a number of psychiatric and neurological disorders, including schizophrenia [6] and depression [7] are related to a chronic inflammatory state and neuroendocrine impairment, which are responsible for a part of their symptomatology. Likewise, AD could be a neuroendocrine disease resulting from an abnormal signaling of insulin that causes the dysfunction of neurons and synaptic processes [8,9]. Cross-sectional clinical studies have shown a positive association between AD and eating behavior [10], particularly with the type of dietary fats [11]. Dyslipidemia, caused by regular consumption of saturated fatty acids (SFA) and trans fatty acids, is positively associated with an increased risk of AD. It has been reported that exposure of tissues to high levels of SFA, particularly palmitic acid, causes the accumulation of cytotoxic lipid intermediates, such as diacylglycerols (DAG) and ceramides; dysfunction of the endoplasmic reticulum (ER) and mitochondria; and oxidative stress and inflammation [12]. Conversely, consumption of fats rich in monounsaturated fatty acids (MUFA) or polyunsaturated fatty acids (PUFA) is associated with a lower prevalence of the disease [10,13], probably as a consequence of lower levels of systemic inflammation [14]. In addition, the MUFA oleic acid increases the flow of fatty acids to the mitochondria and improves their β-oxidation [15,16], attenuating ROS production and the intracellular accumulation of ceramides and DAG [16]. On the one hand, it has been proposed that these beneficial effects depend on the activation of AMP-activated protein kinase (AMPK) [17], which is greatly diminished in the presence of high levels of SFA. Activation of AMPK also reduces ER stress and inflammation, and improves insulin signaling. On the other hand, experiments in rodents fed diets rich in fructose to cause obesity and insulin resistance have shown memory loss and difficulties in learning tasks, which were associated with changes in the structure and function of neurons, and the reduction of the long-term hippocampal enhancement and synaptic plasticity [18,19]. The intake of these diets has also been associated with signs of neuroinflammation, reactive gliosis, and impaired integrity of the BBB [20,21].

Glial cells (astrocytes and microglia) have been identified as critical contributors to the pathophysiological processes associated with neurological diseases, such as schizophrenia and depression, as well as neurodegenerative disorders, such as Parkinson’s and Alzheimer’s diseases [22]. Microglia are protective cells in the central nervous system that are distributed ubiquitously throughout the brain and act as resident macrophages. These cells respond to external adverse stimuli and are activated in order to restore tissue homeostasis [23,24]. Microglial overactivation causes a proinflammatory response, contributing to neuronal damage. As a result of microglial activation, a substantial number of inflammatory mediators are released, including cytokines (TNF-α, IL-1β, IL-6 and IL-10), reactive oxygen species (ROS), and nitric oxide (NO) [25,26]. Concurrently, the endogenous levels of glutathione and the expression of antioxidant enzymes are significantly reduced in patients with AD as compared with healthy controls of the same age [27].

**Figure 1:**
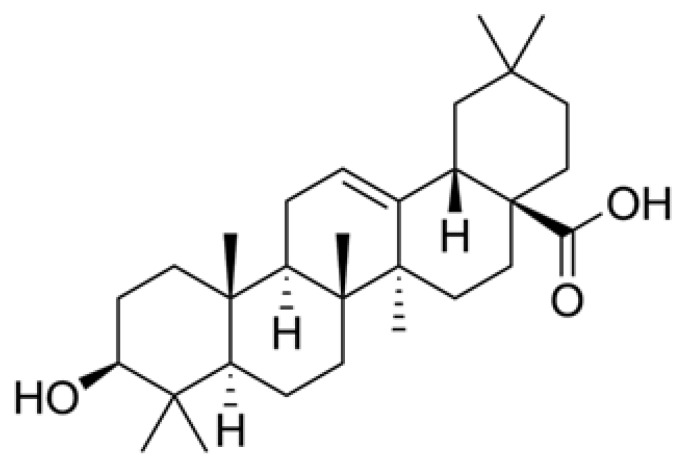
Chemical structure of the oleanolic acid molecule.

To date, there is no curative treatment for AD, so the finding of neuroprotective agents that may help in the prevention or the delay of the disease and relieve its symptoms is a major social and scientific challenge. *In vivo* and *in vitro* studies suggest that anti-inflammatory and antioxidant agents could positively affect the integrity of the BBB and attenuate glial activation in the brains of AD patients [28,29]. In this context, oleanolic acid (3b-hydroxy-olean-12-en-28-oic acid, OA) (Figure 1), a secondary metabolite of plants, appears as an emerging neuroprotective agent [30,31,32,33]. Although it is present in relevant amounts in more than 120 plants [34], few of them have the socioeconomical importance of olive (*Olea europaea* L.). In the olive tree, OA is a key component of the cuticle waxes that cover the fruit and leaf epidermis and is particularly abundant in the leaf, where it can be found at up to 3.5% of the dry weight [35].

Although an officially accepted definition of nutraceuticals is still missing [36,37], OA may be ascribed as a new nutraceutical as it can exert beneficial effects beyond the diet but before the drugs to prevent and treat pathological conditions. In fact, OA can be used as a bioactive component of lipid based functional foods, as we have recently reported after a randomized controlled trial with prediabetic individuals [38]. Clinical testing has been regarded as a prerequisite for the classification of bioactive compounds as nutraceuticals [39].

OA has been proven to have the ability to protect rat cortical neurons from cell death induced by Aβ [30], and to improve the memory deficit also induced by this protein in mice [31]. Likewise, extracts of *Nerium oleander*, which are rich in OA and homologous triterpenes, have exhibited neuroprotection in coronal brain slices from Sprague-Dawley rat pups, where the expression of Nrf2-dependent antioxidant proteins was demonstrated [32]. With this background, and with the aim of unraveling the mechanisms underlying the neuroprotective action of OA in the AD, in this work, we assess the capability of the triterpene to attenuate microglial activation. According to the current definition of an antioxidant [40], OA cannot be considered to be a potent antioxidant, however, it has revealed the ability to modify the expression of key genes in the adaptive cellular response against oxidative stress. OA is an extremely potent enhancer of phase 2 responses, with the potential to stimulate transcription of antioxidant enzymes (superoxide dismutase, catalase, heme oxygenase-1, or peroxiredoxin), as well as genes involved in glutathione (GSH) biosynthesis and regeneration (glutamate-cysteine ligase, glutathione synthase, and glutathione reductase) and in the generation of nicotinamide adenine dinucleotide phosphate (NADPH) (malic enzyme and many of the pentose phosphate pathway). Thus, we hypothesize that OA may exert a neuroprotective effect by mitigating oxidative stress and the release of inflammatory mediators in microglial cells.

In this study, we report for the first time that OA may ameliorate the inflammatory and oxidative responses of microglia upon activation by lipopolysaccharide (LPS). Our findings suggest that OA may be a novel neuroprotective agent that inhibits microglial activation in Alzheimer’s disease.

## 2. Materials and Methods

### 2.1. Materials

Absorbances were measured using a scanning multi-well spectrophotometer (Multiskan spectrum, Thermo Fisher Scientific, Waltham, MA, USA). Fluorescence intensity was measured by a FACSCanto II flow cytometer (Bd Biosciences, San Jose, CA, USA) and calibrating using FACSCanto II analyzer software (Bd Biosciences, San Jose, CA, USA). Gene expression was determined by means of a CFX96 Real-Time PCR system (Bio-Rad, Hercules, CA, USA). DMEM medium, fetal bovine serum (FBS), penicillin, streptomycin, and trypsin were purchased from Biowest (Nuaillé, France). Lipopolysaccharide (LPS) (from E. coli 0111:B4) was purchased from Sigma-Aldrich (St. Louis, MO, USA).

### 2.2. OA Obtaining

High purity OA was obtained from olive tree leaf according to the procedure described by Albi et al. [41]. Briefly, olive tree leaves were extracted by maceration with 96% ethanol (20 mL/g leaf) at room temperature. The ethanolic extract was obtained by filtration and concentrated to induce OA crystallization. OA crystals were separated from the concentrate by filtration and washed with cold 96% ethanol (5 °C to 7 °C), for the elimination of pigments traces and other possible contaminants. Finally, the crystals were submitted to a heat treatment at 165 °C and homogenized to a powder. The OA purity was determined by gas chromatography [42]. Oleanolic acid was identified using a coupled gas chromatography mass spectrometry detector (GC-MS) QP2010 Ultra (Shimadzu Europa GmbH, Duisburg, Germany) fitted with an AOC-20i autosampler, an ion source of electron impact, and a quadrupole detector. OA was determined in an Agilent 6890N GC (Agilent Technologies, CA, USA), equipped with a Rtx-65TG Crossbond capillary column (30 m, 0.25 mm ID, 0.1 mm film thickness) coated with 35% to 65% diphenyl polysiloxane as stationary phase (Restek, Co., Bellefonte, PA, USA) and a FID detector.

### 2.3. Derivatization and Analysis of OA

Derivatization of triterpenic acids is mandatory prior to GC analysis due to their low volatility and high molecular weight. The silylating reagent consisted of a mixture of hexamethyldisilazane, trimethylchlorosilane, and anhydrous pyridine (3:1:9 *v*/*v*/*v*). An aliquot (100 μL) of an OA solution in absolute ethanol (0.5 mg/mL) was placed in an Eppendorf vial, and 100 μL of an ethanolic solution of 18β-glycyrrhetinic acid (0.5 mg/mL) were added as internal standard. The mixture was evaporated to dryness under a N2 stream, the residue immediately dissolved in 200 μL of the silylating reagent and incubated at 80 °C for 2 h. The analysis of silylated OA was performed by GC-MS with a quadrupole detector. The splitless mode was used and the injector temperature was set at 290 °C. Helium as a carrier gas at a pressure of 53.1 kPa and a flow of 1 mL/min was used. The oven temperature program was as follows: initial temperature, 50 °C for 1min; 50–200 °C at 40 °C/min; 200–280 °C at 10 °C/min; and finally held for 2 min. The total run time was 14.75 min. The MS conditions were as follows: interface temperature, 280 °C; ion source temperature, 220 °C; electron impact, 70 eV; and acquisition mode, scan (*m*/*z* 50 to 600). The identification of OA was accomplished by comparing the retention times and abundance ratios of two fragment ions (203 and 189 *m*/*z*).

### 2.4. GC-FID Determination of OA

An aliquot (1 μL) of the silylated sample was determined by GC-FID. The injection was realized in split mode, and hydrogen was used as carrier gas (pressure at column head 140 kPa). The oven temperature was initially established at 260 °C for 9 min, programmed to increase up to 280 °C at a rate of 10 °C/min and maintained at this value for 8 min. The injector and detector temperatures were isothermally established at 300 °C. The retention time for OA under these chromatographic conditions was 7.7 min.

### 2.5. Cell Line

The mouse BV2 cell line (C57BL6) (http://bioinformatics.hsanmartino.it/cldb/cl7130.html) was used as a microglial cell model. BV2 cells have morphological, phenotypic, and functional markers of macrophages. The cells were generously provided by Alberto Pascual (Institute of Biomedicine of Seville, IBIS, Seville, Spain), and cultured in DMEM medium supplemented with 10% heat-inactivated fetal bovine serum (FBS) and antibiotics (100 U/mL penicillin and 100 U/mL streptomycin), under a 100% humidified atmosphere of air +5% CO_2_ at 37 °C. Cells were passaged every 2 to 3 days to maintain growth. The experiments to assess the effect of OA on the microglial phenotype and gene expression were carried out in medium reduced to 5% FBS.

### 2.6. Cell Viability Assay

**Figure 2:**
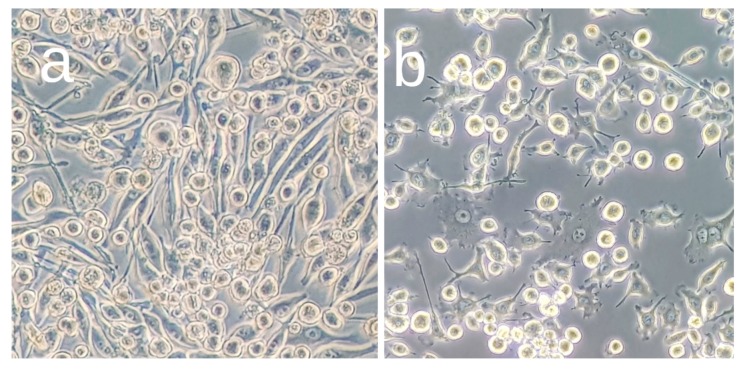
Microphotographs of BV2 microglial cells before (**a**) and after (**b**) activation with lipopolysaccharide (LPS).

The effect of the OA application on the cell viability was evaluated by the 3-(4, 5-dimethylthiazol-2-yl)-2, 5-diphenyl tetrazolium bromide (MTT) assay, which is based on the cleavage of the yellow tetrazolium salt MTT to purple formazan crystals by metabolic active cells [43]. The BV2 cells were seeded in a 96-well plate at a density of 5 × 10^5^ cells per well and pretreated with various concentrations of OA (0.5 to 25 μM) or vehicle (dimethyl sulfoxide, DMSO) for 1 h, followed by treatment with LPS 1 μg/mL to induce activation (Figure 2) for 24 h at 37 °C, to trigger the microglial activation. The assay was performed following the instructions of the manufacturer’s kit (Sigma, St. Louis, MO, USA), and the resulting colored solution was quantified by measuring absorbance at 570 nm. Cell viability was calculated using the equation: (mean OD treated cells/mean OD control cells) × 100.

### 2.7. Inflammatory Cytokines Production

BV2 cells were seeded at 5 × 10^5^ cells/mL in 6-well plates and pretreated with OA (0.5 to 10 μM) for 1 h, followed by LPS (100 ng/mL) for 24 h at 37 °C. Culture media were collected and the IL-6, IL-1β, and TNF-α production measured using ELISA kits (Diaclone, Besancon, France) according to the manufacturer’s instructions. Absorbance was read at 450 nm.

### 2.8. Reactive Oxygen Species Determination

For the determination of the intracellular concentration of ROS, the fluorescent probe, 2′,7′-dichlorofluorescin diacetate (DCFDA), a fluorogenic dye that measures hydroxyl, peroxyl, and other ROS activity within the cell was used. After diffusion into the cell, DCFDA was deacetylated by cellular esterases to a nonfluorescent compound, which was later oxidized by ROS into 2′,7′-dichlorofluorescein (DCF), a highly fluorescent compound that was quantified by flow cytometry at 485 nm (excitation) and 535 nm (emission) [44].

### 2.9. Nitric Oxide Assay

BV2 cells were seeded in a 96-well plate at a density of 5 × 10^5^ cells per well and incubated overnight. Cells were pretreated with various concentrations of OA (0.5 to 10 μM) for 1 h, and, then, with LPS (1 μg/mL) for 24 h at 37 °C. After the treatments, culture supernatants were collected, and the NO concentration was measured using a Griess reagent assay kit (R&D Systems, Inc. Minneapolis, MN, USA), which measures the level of accumulated nitrite, a NO metabolite. Absorbance was determined at 540 nm.

### 2.10. Glutathione Assay

The culture supernatants after the OA and LPS treatments were also collected to analyze the total glutathione levels by using a modification of the glutathione reductase recycling assay [45]. Reduced glutathione (GSH) was oxidized by 5,5′-dithiobis-(2-nitrobenzoic acid) (DTNB) resulting in the formation of oxidized glutathione (GSSG) and 5-thio-2-nitrobenzoic acid (TNB). Then, GSSG was reduced to GSH by glutathione reductase (GR) using reducing equivalents provided by NADPH. The rate of TNB formation was proportional to the sum of GSH and GSSG present in the cell culture sample and was determined at 412 nm.

### 2.11. Gene Expression

Gene expression was measured by reverse transcription quantitative polymerase chain reaction (RT-qPCR). The BV2 cells were seeded at 5 × 10^5^ cells/mL in 6-well plates and pretreated with OA (0.5-10 μM) for 1 h, followed by LPS (100 ng/mL) for 24 h at 37 °C. Total RNA was isolated using Trisure (Bioline, Meridian Life Science, Inc. Memphis, USA), and reverse transcribed to cDNA using a synthesis kit (NZYTech, Lisboa, Portugal). Quantitative PCR was performed by means of a Precision Plus Master Mix kit containing SYBR Green (PrimerDesign Ltd., Chandler’s Ford, UK), according to the manufacturer’s instructions and the following conditions: Denaturation at 95 °C for 2 min, followed by 40 cycles at 95 °C for 15 s and at 60 °C for 60 s. The relative gene expression was quantified according to the 2^−ΔΔCt^ method, and the results were expressed as fold difference with the control (no LPS stimulation), after normalization with hypoxanthine phosphoribosyl-transferase (HPRT). The following mouse primer sequences were used: IL-1 β, forward GACCTTCCAGGATGAGGACA, reverse AGCTCATATGGGTCCGACAG; IL-6, forward AGTTGCCTTCTTGGGACTGA, reverse TCCACGATTTCCCAGAGAAC; TNF-α, forward AGTCCGGGCAGGTCTACTTT, reverse GAGTTGGACCCTGAGCCATA; inducible nitric oxide synthase (iNOS), forward CTCACTGGGACAGCACAGAA, reverse GGTCAAACTCTTGGGGTTCA; and HPRT, forward TGCTCGAGATGTCATGAAGG, reverse TATGTCCCCCGTTGACTGAT.

### 2.12. Statistical Analysis

All experiments were performed in triplicate and data expressed as mean ± standard deviation (SD) of three independent experiments and analyzed using the IBM SPSS Statistics 23.0 (IBM Corp., Armonk, NY, USA) software. Comparisons between control and treatment groups were assessed using ANOVA followed by Tukey’s test. The significant difference was set at *, *p* < 0.05; **, *p* < 0.01; and ***, *p* < 0.001.

## 3. Results

### 3.1. Effects of OA Pretreatment on BV2 Cell Viability

The MTT assay was performed to determine cell viability when treated with OA. Viability of BV2 cells was compromised when incubated with OA concentrations greater than 25 μM for 24 h. This treatment reduced the number of metabolically active cells to 40% of the initial cell population (data not shown). In contrast, cell viability was not threatened by the presence of OA concentrations lower than 10 μM, a range in which the proportion of active cells always remained above 85%. It was found that both DMSO and LPS, at the applied concentrations, did not affect the viability of BV2 cells. Therefore, the subsequent experiments were performed considering OA concentrations in the range 0.5 to 10.0 μM.

### 3.2. OA Reduces Production and Expression of Proinflammatory Mediators in LPS-Stimulated BV2 Cells

**Figure 3:**
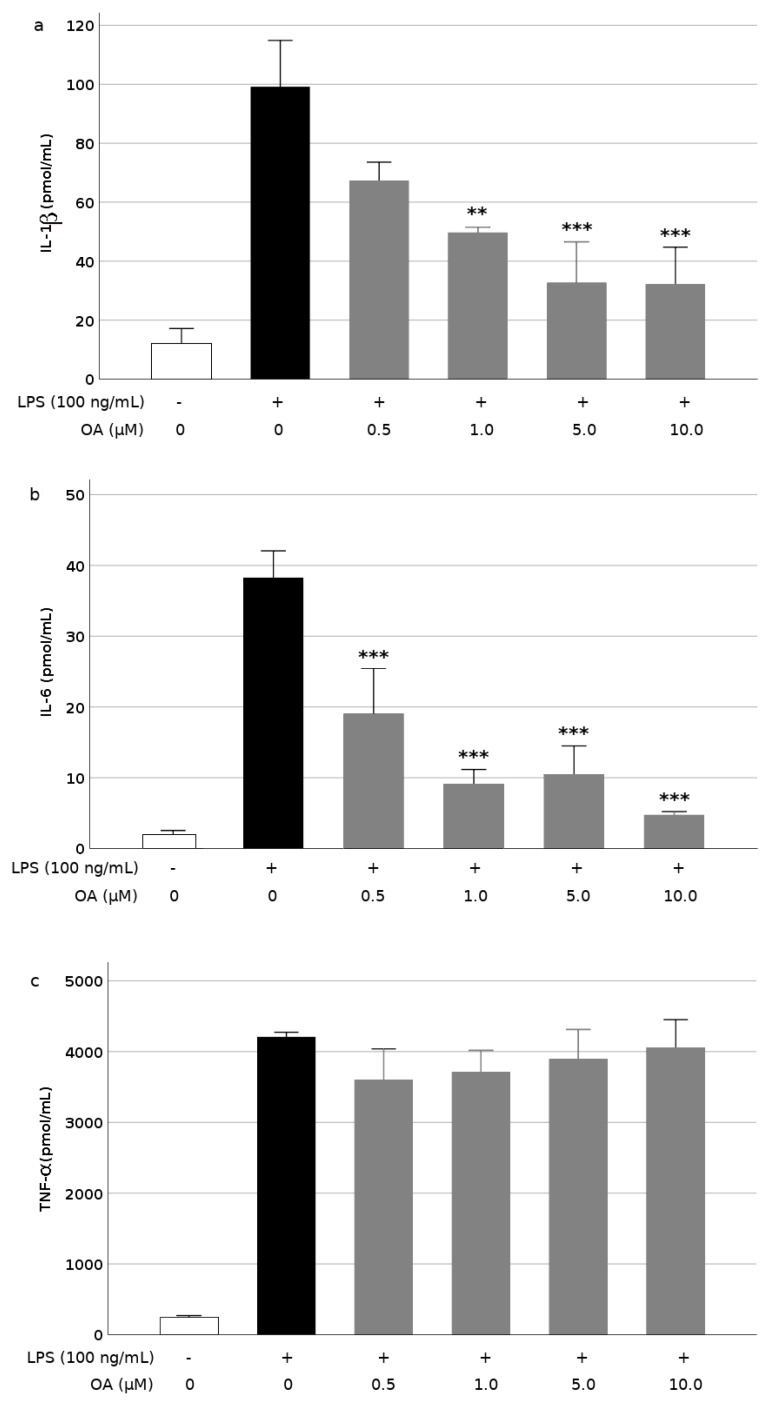
Inhibition of cytokine production by oleanolic acid (OA) in LPS-induced microglia. (**a**) interleukin-1β (IL-1β), (**b**) interleukin-6 (IL-6), and (**c**) tumor necrosis factor-α (TNF-α). Cells were pretreated with OA for 1 h before LPS treatment for 24 h. Values are expressed as mean ± SD of 3 independent experiments. **, *p* < 0.01 and ***, *p* < 0.01 vs. LPS.

The release and gene expression of proinflammatory mediators were measured to determine BV2 cell activation after stimulation with LPS and the attenuation caused by pretreatment with OA. Our results also show that OA exerts a protective action against these processes associated with microglial activation. The treatment of BV2 cells with LPS caused their activation, as revealed by a burst in the production of the inflammatory cytokines IL-1β, IL-6, and TNF-α (Figure 3), however, pretreatment of cells with OA attenuated this LPS-induced response. The OA effect was dose-dependent for IL-1β and IL-6 production. A significant inhibition was observed at doses of 1.0 to 10 μM OA for IL-1β (Figure 3a) and at all doses for IL-6 (Figure 3b). OA reduced IL-1β release by the BV2 cells by 30% at the lowest concentration and by 70% at the highest concentration assayed. Regarding IL-6, reductions ranged from 50%–88% at the lowest and highest concentration, respectively. In contrast, TNF-α release was not significantly affected by OA-pretreatment, although modest reductions, between 5% and 15%, were observed (Figure 3c).

**Figure 4:**
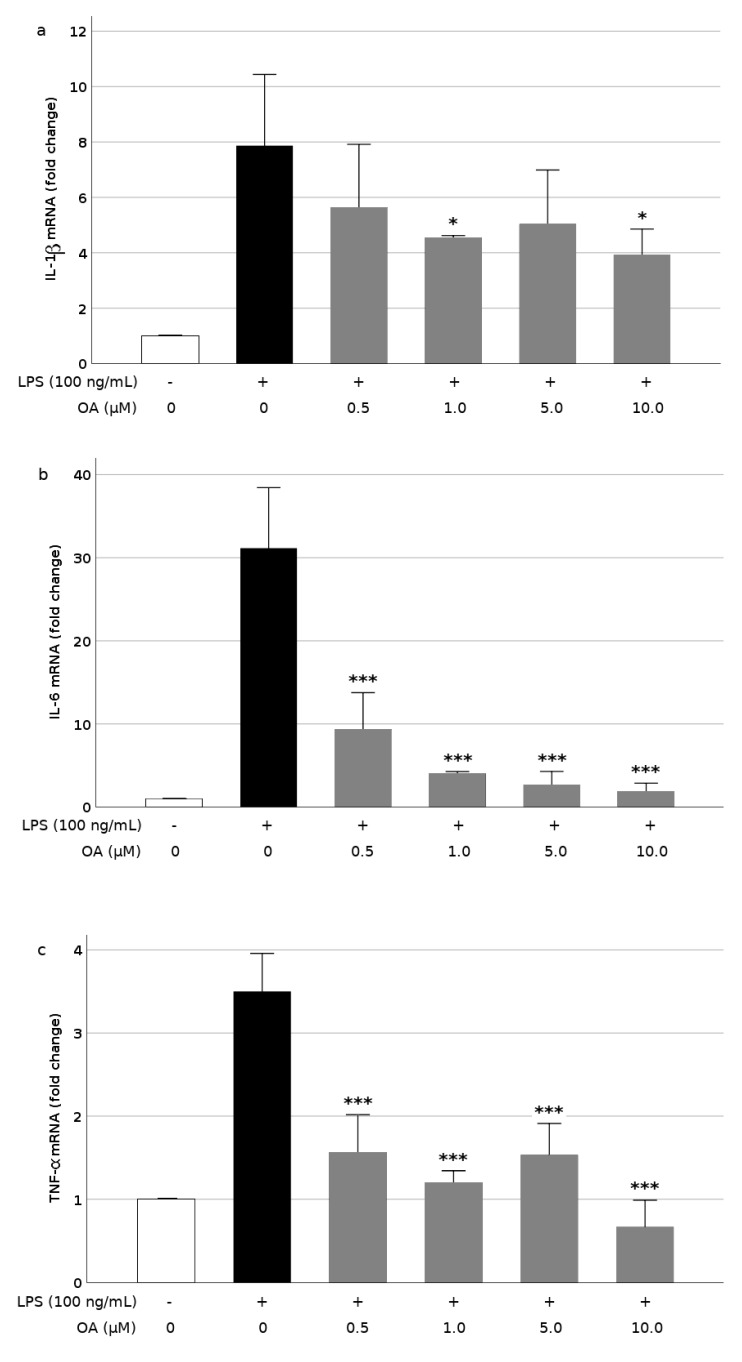
Inhibition of cytokine gene expression by oleanolic acid (OA) in LPS-induced microglia: (**a**) interleukin-1β (IL-1β), (**b**) interleukin-6 (IL-6), and (**c**) tumor necorsis factor-α (TNF-α). Cells were pretreated with OA for 1 h before LPS treatment for 24 h. Values are expressed as mean ± SD of 3 independent experiments. *, *p* < 0.05 and ***, *p* < 0.001 vs. LPS.

To further study the mechanisms underlying cytokine release, we measured the gene expression of these mediators in LPS-stimulated BV2 cells. The burst in IL-1β, IL-6, and TNF-α caused by LPS was due, at least partially, to an augmented *de novo* synthesis of these proteins, because the expression of their encoding genes experienced important increases, however, preincubations with OA (0.5 to 10.0 μM) reduced significantly the LPS-induced expression of those genes, although at different extensions. The IL-1β expression was depressed between 28% at 0.5 μM OA and 50% at 10 μM (Figure 4a). In the case of IL-6, a stronger and dose-dependent downregulation, between 70% and 93% was observed at the lowest and highest concentration assayed (Figure 4b). Finally, the LPS-induced TNF-α expression was reduced between 56% and 82% by pretreatment with OA (Figure 4c). This contrasts with the apparent little affectation of TNF-α levels by OA.

### 3.3. OA Reduces Production of NO and Expression of iNOS in LPS-Stimulated BV2 Cells

**Figure 5:**
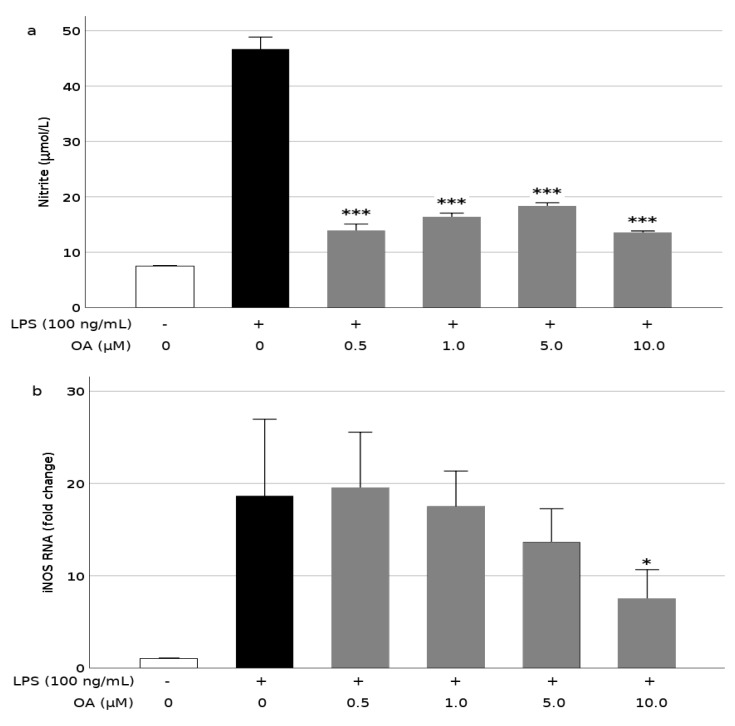
Inhibition of nitrite production (**a**) and inducible nitric oxide synthase (iNOS) gene expression (**b**) by oleanolic acid in LPS-induced microglia. Cells were pretreated with OA 1 h before LPS treatment for 24 h. Values are expressed as mean ± SD of 3 independent experiments. *, *p* < 0.05 and ***, *p* < 0.001 vs. LPS.

We also investigated the effect of OA on NO production, which is regulated by iNOS activity. On the one hand, stimulation of the BV2 cells with LPS induced a seven-fold increase in NO release, as indicated by the determination of nitrite with the Griess reagent (Figure 5a), however, preincubation with OA prevented this rise, and NO production was repressed between 60% and 70%. At least in the 0.5–10.0 μM range, this effect was not dose-dependent. On the other hand, the decline of LPS-induced NO production caused by the OA pretreatment did not seem totally derived from a repression of iNOS at the translational level, because its gene expression was not significantly affected by OA, except at the highest concentration of the triterpene (Figure 5b). At that concentration (10.0 μM), iNOS expression was reduced by more than 50%.

### 3.4. OA Reduces ROS Release in LPS-Stimulated BV2 Cells

**Figure 6:**
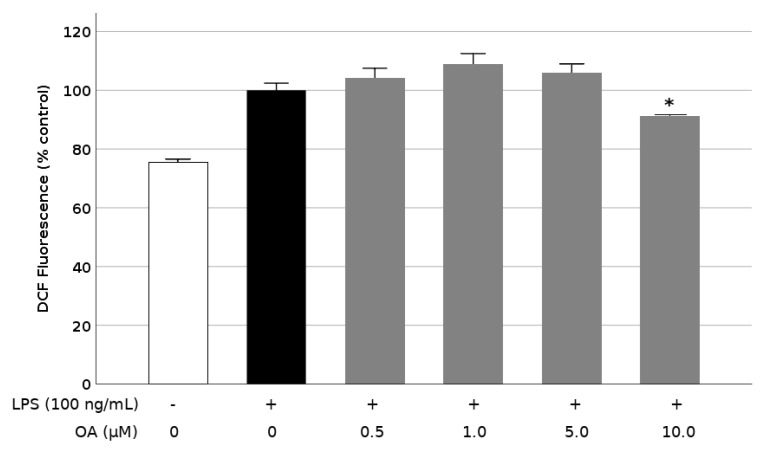
Inhibition of reactive oxygen species (ROS) production by oleanolic acid (OA) in LPS-induced microglia. Cells were pretreated with OA for 1 h before LPS treatment for 24 h. Data are presented as percentage of DCF production. Values are expressed as mean ± SD of 3 independent experiments. *, *p* < 0.05 vs. LPS.

Since Alzheimer’s disease is associated with extensive oxidative stress, we studied the release of ROS by the BV2 cells exposed to LPS in the culture medium, using the fluorescent probe DCFDA and flow cytometry. Treatment of cells with 100 ng/mL LPS caused a 33% increase in the intracellular production of ROS. The presence of OA had little effect on this moderate rise in radical species, because the triterpene was only able to reduce the LPS-induced ROS production by a modest 9% when it was applied at 10 μM (Figure 6). Within the 0.5–5.0 μM range, OA had no ostensible influence on intracellular ROS production.

### 3.5. OA Increases Production of Glutathione in LPS-Stimulated BV2 Cells

**Figure 7:**
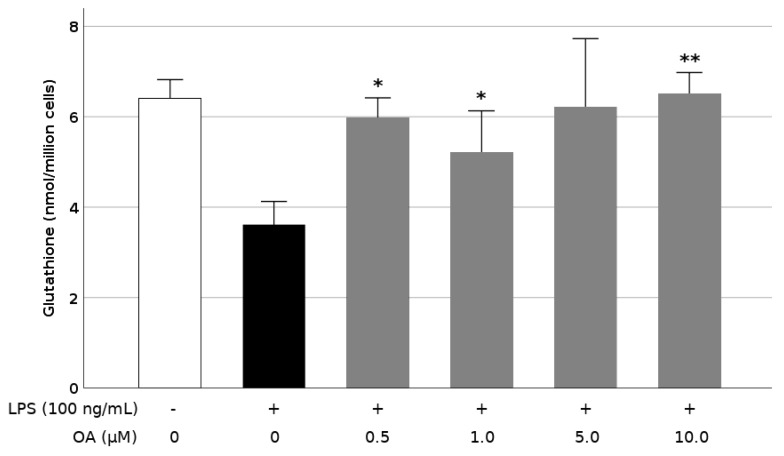
Increased total intracellular glutathione concentration by oleanolic acid (OA) in LPS-induced microglia. Cells were pretreated with OA for 1 h before LPS treatment for 24 h. Values are expressed as mean ± SD of 3 independent experiments. *, *p* < 0.05 and **, *p* < 0.01 vs. LPS.

It has been reported that the endogenous antioxidant system in microglia is depressed during cell activation. To evaluate this feature as it relates to the neuroprotective role of OA, we investigated the evolution of the intracellular GSH content in the LPS-stimulated BV2 cells, in the absence and presence of the triterpene. Stimulation with 100 ng/mL LPS reduced the glutathione level up to 44% as compared with cells treated only with vehicle (DMSO) (Figure 7). Nevertheless, preincubation of cells with 0.5 to 10.0 μM OA restored the baseline levels of GSH, demonstrating a reinforcement of the cell antioxidant response by the triterpene. We found significant increments in GSH at all OA concentrations, except 5.0 μM. The lack of significance at that concentration was probably due to a high variability of the results. In any case, GSH levels were increased by almost twice after pretreatment with OA as compared with LPS alone.

## 4. Discussion

Microglia plays a crucial role in both brain protection and neurodegenerative diseases. By acting as resident macrophages, these cells are activated after detecting alterations in brain homeostasis [23,24]. Under physiological circumstances, they participate in controlled immune reactions critical for neuronal survival. However, under pathophysiological conditions, their exposure to misfolded proteins (as Aβ or hyperphosphorylated tau) and signals of neuronal damage lead to an exacerbated activity, which causes the chronic production of radical species and proinflammatory cytokines. A positive feedback loop between neuronal injury and inflammation is built, which triggers neuronal death pathways and activates other glial cells [1,46,47]. For this reason, microglial activation has been proposed as a therapeutic target for neurodegenerative diseases. In experimental models, activation of microglia may be induced by different cell insults, being LPS, an endotoxin of Gram-negative bacteria, one of the most used. Activation of BV2 cells with LPS releases abundant amounts of ROS and NO, as well as TNF-α, IL-1β, and IL-6.

In this study, we report for the first time that direct treatment of microglial cells with OA confers cytoprotection against these effects. We have demonstrated that OA strongly represses the LPS-induced production of IL-1β and IL-6 in a dose-dependent manner, and reduces the gene expression of TNF-α.

The generation of radical species and oxidative stress underlies microglial activation. Indeed, the stimulation of BV2 cells with LPS increased the intracellular production of NO and ROS. These events were alleviated to some extent by pretreatment with OA. An excessive NO production has been associated with chronic inflammation in neurodegenerative diseases. NO is a small gaseous molecule, that easily crosses cell membranes, which at normal physiological levels acts as a neurotransmitter at synaptic junctions, however, high levels of NO secreted by activated microglia can lead to the formation of peroxynitrite (ONOO^−^), contributing to lipid peroxidation, ROS production, protein and mitochondrial damages, DNA oxidation, and eventually to neuronal damage [48]. NO is synthesized from L-arginine in a reaction catalyzed by NO synthases (NOS) [49]. The inducible isoform of NOS (iNOS) is induced in microglia in response to inflammatory mediators. Hence, the assessment of NO production is relevant to understand microglial-induced inflammation. Our present data show that both NO production and iNOS expression in BV2 cells were markedly increased after exposure to LPS, however, they were reduced by pretreatment of these cells with OA. In this case, NO production was blocked by OA more severely than iNOS gene expression, suggesting a possible post-translational effect of the triterpene. On the other hand, OA seems to have no ostensible influence on ROS levels.

Different studies have proposed that antioxidant agents may attenuate microglial activation by modulating the intracellular ROS levels [50]. In a previous work, we showed that OA possesses a limited ability to directly scavenge radical species, such as peroxyl, 2,2′-azino-bis(3-ethylbenzothiazoline-6-sulphonic acid) (ABTS); 2,2-Diphenyl-1-picrylhydrazyl (DPPH); and 2,2′-azobis (2-amidinopropane) dihydrochloride (AAPH) [51]. However, this apparent weakness is compensated with a potent activity as an inductor of phase II responses, eliciting the reinforcement of the adaptive cell defense against oxidative and chemotoxic stresses. Many of these OA effects seem mediated by the nuclear factor erythroid 2-related factor 2 (Nrf2) [52], which binds to antioxidant response elements in the gene promoter region and stimulates transcription of antioxidant enzymes (SOD, CAT, heme oxygenase-1, glutathione peroxidase, or peroxiredoxin), as well as genes involved in GSH biosynthesis (glutamatecysteine ligase and glutathione synthase) and regeneration (glutathione reductase), among others. In fact, in this study, we have corroborated that OA restored the levels of glutathione, which were depressed in microglial cells by the exposure to LPS. We have also reported that OA can activate directly Nrf2 at the protein level via the Michael addition reaction with Keap1, the primary sensor that retains Nrf2 in cytoplasm for ubiquitin-dependent degradation [53], but also indirectly through stimulating of stress-induced signaling pathways, such as the transduction cascades mediated by MAPK (ERK and JNK) and AMPK [52].

The data shown in this study are consistent with other previously reported for OA and related triterpenoids. Matumba et al. [54] described a similar response to OA in rats fed a high-fructose diet, showing that the effect of OA on the repression of TNF-α levels in skeletal muscle cells was more modest than that on gene expression. Fan et al. [55] investigated the potential of hyperoside, a galactoside of quercetin, to inhibit inflammatory mediators in BV2 microglial cells stimulated with LPS. They showed that hyperoside significantly inhibited production of NO and proinflammatory cytokines, including IL-1β and TNF-α, as well as the expression of iNOS. Moreover, they observed that the effect of the hyperoside pretreatment on TNF-α levels in LPS-induced BV2 cells was low as compared with the effects on IL-1β and IL-6. β-Amyrin, a biosynthetic precursor and structural analog of OA, has been demonstrated to exert a potent inhibitory effect on the production of inflammatory factors (PGE2, TNF-α, and IL-6) in RAW 246.7 macrophages stimulated with LPS [56]. Furthermore, da Silva et al. [57] showed that β-amyrin inhibited the production and release of the proinflammatory cytokines TNF-α, IL-1β, and IL-6 in complete Freund’s adjuvant-treated mice; and attributed these effects to the activation of cannabinoid receptors. Induction of cannabinoid receptors activates MAPK cascade [58]. Some natural phytochemicals, defined as phytocannabinoids, can bind to cannabinoid receptors and promote anti-inflammatory effects. In addition, Cha et al. [59] pointed out that the anti-inflammatory mechanisms of oleanane-type pentacyclic triterpenes could resemble that of glucocorticoids, because of their structural similarity.

Papyriogenin D, an oleanane-type triterpene isolated from Tetrapanax papyriferus, showed significant NO inhibitory activity in BV2 cells, reducing the LPS-induced expression of COX-2 and proinflammatory cytokines, such as TNF-α and IL-6 [60]. Likewise, Liu et al. [61] reported that pretreatment of BV2 cells with paroxetine significantly inhibited the LPS-induced production of NO, as well as TNF-α and IL-1β. The expression of iNOS and cytokine genes was also attenuated. These authors investigated the operating signaling pathways and revealed that the paroxetine-mediated inhibition of NO production occurred via suppression of LPS-induced JNK1/2 activation, whereas the diminishing of cytokine levels proceeded via downregulation of JNK1/2 and ERK1/2 pathways. These MAPK enzymes were responsive to stress stimuli, including cytokines. Similarly, improvements in the intracellular glutathione pool and in the activity of catalase and SOD enzymes by pretreatment with OA have been reported in PC12 cells, a cell line derived from a pheochromocytoma of rat adrenal medulla, exposed to H_2_O_2_ and 1-methyl-4- phenylpyridinium [62]. These effects of OA were mediated through activation of Nrf2 [63].

Many inflammatory mediators, including cytokines and NO, are regulated by nuclear factor κ B (NFκB), which is a key player in controlling both innate and adaptive immunities [64]. NFκB is present in the cytoplasm in association with the inhibitory protein IκB. After activation by different inducers (ROS, TNFα, IL-1β, or bacterial LPS), IκB becomes phosphorylated and degraded by the proteasome, allowing NFκB to translocate to the nucleus and bind specific ADN sites to regulate the transcription of a large number of genes, including cytokines, chemokines, and stress-response proteins. The constitutive activation of NFκB pathways is associated with inflammatory diseases. Different studies have demonstrated that OA and homologous triterpenes are potent inhibitors of IKK, and therefore they block NFκB activation -. By doing that, OA reduces TNFα-induced E-selectin expression in human endothelial cells [65]. IL-6 release in LPS-activated MonoMac-6 cells [66], and endothelin-1pathway in Zucker diabetic rats [67]. Hence, it seems clear that OA may dampen both oxidative stress and inflammatory response by using its ability to act on the Nrf2 and NFκB pathways. Because several anti-inflammatory agents that suppress NFκB signaling also activate the Nfr2-ARE cascade and, as it has been pointed out, NFκB participates in the transcriptional regulation of Nrf2, the existence of a competitive cross-talk relationship with each other has been postulated [68,69].

This work has some limitations. There is an in vitro study on microglial cells, so conclusions cannot be extended to other cells or whole organisms. A common feature of in vitro studies is that the doses of the bioactive compounds assayed cannot be transposed from actual intakes in animals or human beings but are adapted from the results of cell viability assays. However, the results presented here are a solid pillar for future in vivo studies and clinical trials. In addition, we did not determine the expression of NfκB and Nrf2, together with other signaling proteins that might be involved in the effect of OA on microglial activation. Investigation of the signaling pathways and metabolic routes in which OA exerts it effect deserves a profound study that should be performed in the future.

## 5. Conclusions

**Figure 8:**
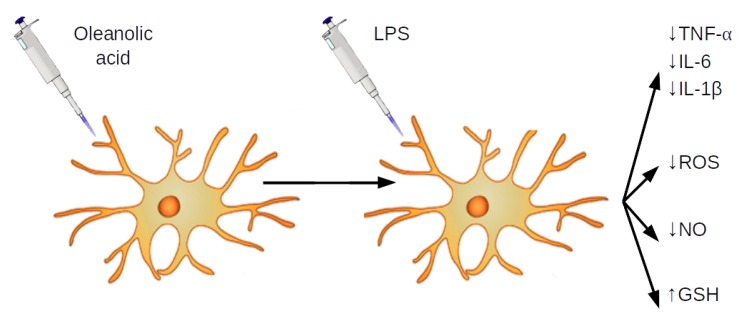
Graphical summary of the main results obtained.

In conclusion, in this study, we report for the first time that OA may ameliorate the inflammatory and oxidative responses of microglia upon activation by LPS. Figure 8 shows a graphical summary of the main findings. In brief, OA reduced the release of IL-1β, IL-6, TNF-α, and NO and caused a modest inhibition of ROS production. These effects of the triterpene were associated with the downregulation of genes encoding for cytokines and iNOS, and the restitution of the glutathione levels. We suggest that these OA actions are derived from its interaction with the transcription factors, Nrf2 and NfκB, and the reinforcement of the adaptive cell response against oxidative stress and inflammation. Our findings, in this study, advocate that OA could be considered a novel neuroprotective agent to inhibit microglial activation in neurodegenerative diseases, such as Alzheimer’s disease, however, further studies are required to fully elucidate the molecular mechanisms that are involved in these actions, and therefore exploit the therapeutic potential of this natural triterpene.
